# The Harbin Medical University nephrectomy score: a quantitative system for evaluating the complexity of laparoscopic retroperitoneal simple nephrectomy

**DOI:** 10.1590/S1677-5538.IBJU.2018.0634

**Published:** 2019-12-17

**Authors:** Yiwen Liu, Chunyang Wang, Xiuhai Wu, Linglong Kong, Shaobin Ni

**Affiliations:** 1 Department of Urology, The First Affiliated Hospital of Harbin Medical University, Harbin, Heilongjiang, P.R. China

**Keywords:** Laparoscopy, Nephrectomy, Surgical Procedures, Operative

## Abstract

**Background:**

Laparoscopic retroperitoneal simple nephrectomy (LRSN) has been widely accepted as a mainstay option for benign non-functioning kidney. The complexity of the procedure, however, differs and remains a subject of controversy.

**Objective:**

To develop a standardised Harbin Medical University nephrectomy score (HMUNS) system for evaluating LRSN complexity.

**Subjects and methods:**

A total of 6 variables with different factors comprising primary diseases, history of upper urinary tract surgery, body mass index (BMI), surgeon's learning curve, kidney volume, and Mayo Adhesive Probability (MAP) scores were included in the HMUN score. 95 consecutive patients who underwent LRSN at our institution were divided into low (2 to 6 points) and high (7 to 17 points) complexity groups with HMUNS and investigated the differences of operative time (OT), estimated blood loss (EBL), postoperative hospitalisation time (PHT), rate of intraoperative conversion to open surgery, and the Clavien-Dindo classification (CDC) between both groups.

**Results:**

Longer mean operative times (193.2±69.3 min vs. 151.9±46.3 min, p <0.05), more median estimated blood loss (100.0mL vs. 50.0mL, p <0.05), and higher rates of conversion to open surgery (1.2% vs. 25%, p <0.05) were observed in the high-complexity group (n=12) than in the low-complexity group (n=83). However, there were no remarkable differences between the two groups related to the baseline characteristics, post-surgical hospitalisation times, and postoperative complications.

**Conclusions:**

The HMUNS can effectively reflect LRSN complexity, thus providing a quantitative system for risk estimation and treatment decisions. Because of some limitations, further well-designed studies are necessary to confirm our findings.

**Patient summary:**

The HMUNS, including primary diseases, history of upper urinary tract surgery, BMI, surgeon's learning curve, kidney volume, and MAP score, can provide an effective quantitative tool to evaluate the complexity of LRSN.

## INTRODUCTION

Laparoscopic techniques have been increasingly employed for the removal of benign non-functioning kidneys since 1991 (1) due to apparent advantages such as less blood loss, pain, and hospitalisation time than open surgical procedures (2). Laparoscopy can be performed either transperitoneally or retroperitoneally depending on the anatomic characteristics of the diseases and the surgeon's experience. Although no notable differences have been observed regarding clinical efficacy and safety, the retroperitoneal approach remains an option of choice for less involvement of the intraperitoneal organs and more direct access to the renal arteries (3). Given that the indications or contradictions of laparoscopic retroperitoneal simple nephrectomy (LRSN) remain controversial, a reproducible and comprehensive scoring system merits elaborate investigations. Therefore, we proposed and developed a standardised Harbin Medical University Nephrectomy Score (HMUNS) to assess the complexity and risk of LRSN based on our data.

## MATERIAL AND METHODS

### The HMUNS system

The HMUNS system, as summarised in Table-1, consists of six reproducible elements pertinent to LRSN feasibility reviewed from the available literature. Each element was assigned different points on the basis of its relevant features. Primary diseases (2, 3) such as renal tuberculosis or pyonephrosis were assigned 2 points and autosomal dominant polycystic kidney disease (ADPKD) was assigned 3 points. For those with a history of upper urinary tract surgery (0-3), cases with no history of urinary surgery were assigned 0, while those involving extracorporeal shock wave lithotripsy (ESWL), ureterorenoscopy/percutaneous nephrolithotomy (URS/PCNL), and retroperitoneal surgery were assigned 1, 2, and 3 points, respectively. For body mass index (BMI) (0-3), 0 points were given to BMI <25, 1 point was awarded to BMI ≥25 but <30, 2 points were assigned to BMI ≥30 but <35, and 3 points were given to BMI ≥35. For the surgeon's learning curve (1–2), the number of surgical experiences greater than 30 cases was deemed relatively easy for surgeons, while fewer than 30 cases was considered relatively difficult; therefore, 1 and 2 points were assigned to <30 cases and ≥30, respectively. Renal volume (1–3) was calculated using the formula kidney volume (KV)=π/6×renal length (L)×renal width (W)×renal depth (D) (4), and scores of 1, 2, and 3 points were assigned when it was <500cm^3^, 500-1000cm^3^, and ≥ 1000cm^3^, respectively. For the Mayo Adhesive Probability score (5) (0-3), no stranding (type 1), thin and mild stranding (type 2), and diffuse and thick-banded severe stranding (type 3) in CT were assigned 0, 2, and 3 points, respectively. The combined HMUN scores varied from 2 to 17 points. For further evaluation, each LRSN case was graded as low complexity (2-6 points) or high complexity (7-17 points).

**Table 1:** The Harbin Medical University Nephrectomy Score.

	0 point	1 point	2 points	3 points
Primary disease	–	–	Tuberculosis; Pyonephrosis	ADPKD
History of upper urinary surgery	none	ESWL	URS, PCNL	Retroperitoneal surgery
BMI	BMI < 25	25 ≤ BM I< 30	30 ≤ BMI< 35	BMI ≥ 35
Learning curve	–	≥ 30 cases	< 30 cases	–
Renal volume	–	< 500 cm^3^	500-1000 cm^3^	> 1000 cm^3^
Mayo Adhesive Probability score	No stranding	–	Thin and mild stranding	Diffuse, thick-banded stranding

**ADPKD** = Autosomal Dominant Polycystic Kidney Disease; **ESWL** = extracorporeal shock wave lithotripsy; **URSL** = Ureteroscopic; **PCNL** = percutaneous nephrolithotomy; **BMI** = body mass index

### Patient's baseline characteristics and perioperative variables

The consecutive records of patients who underwent LRSN for various benign diseases at our institution, the Department of Urology at the First Affiliated Hospital of Harbin Medical University, were reviewed retrospectively from January 2013 to December 2017. All patients were diagnosed with non-functioning kidneys with a split renal function of <10% before surgery by nuclear renal function studies (6). All cases were classified into two complexity groups according to the HMUNS, and the imaging data including CT (computed tomography) and ultrasonography were evaluated by two individual reviewers. The endpoints consisted of operative time (OT), estimated blood loss (EBL), postoperative hospitalisation time (PHT), rate of intraoperative conversion to open surgery, and postoperative complications stratified using the Clavien-Dindo classification (CDC) (7). Our study was approved by the institutional review board of our institution and conducted in accordance with the principles of the Declaration of Helsinki (8).

#### Surgical techniques

The patients were placed in the lateral flank position with elevation of the diseased kidney bridge after a tracheal intubation for general anaesthesia. Inside-out sterilisation of the skin was performed with a reasonable range and draping of the operative area in a regular sequence. Three trocars were employed in all of the cases. A 1.5- to 2cm incision was made under the twelfth rib on the posterior axillary line. Then, we bluntly penetrated the muscle layer and lumbodorsal fascia via forceps to reach the retroperitoneal space, which was dilated using an index finger and the balloon successively. Then, 12mm and 10mm trocars were placed at the level of 1-2cm above the crista iliaca on the mid-axillary line and the anterior axillary line around the eleventh rib tip, respectively. After dissecting and identifying the medial edge of the psoas muscle and peritoneum, a longitudinal incision in Gerota's fascia parallel to the psoas muscle was cut using an ultrasonic scalpel. It should be noted that the peritoneal organs were close and susceptible to injury. The renal hilum was accessed using a blunt and/or sharp dissection, and the renal artery and vein were ligated and dissected with three Hem-o-lok clips, respectively. Sequentially, the mobilisation of the kidney was performed after dissection of the ureter. The excised kidney and proximal ureter were removed en bloc by extending the twelfth rib incisions to 7-10cm Gibson incisions.

#### Statistical analysis

The Wilcoxon signed-rank test was utilised to analyse the statistical data of the EBL and PHT between the two groups, while the two-sample t-test was used to analyse OT. A Chi-squared test was employed to assess the statistical differences in the rate of conversion to open surgery as well as the postoperative complications (CDC). All analyses were performed using IBM SPSS Statistics 24 software (www.sciencesoftware.com.cn), and the differences were deemed significant when p <0.05.

## RESULTS

**Figure 1:**
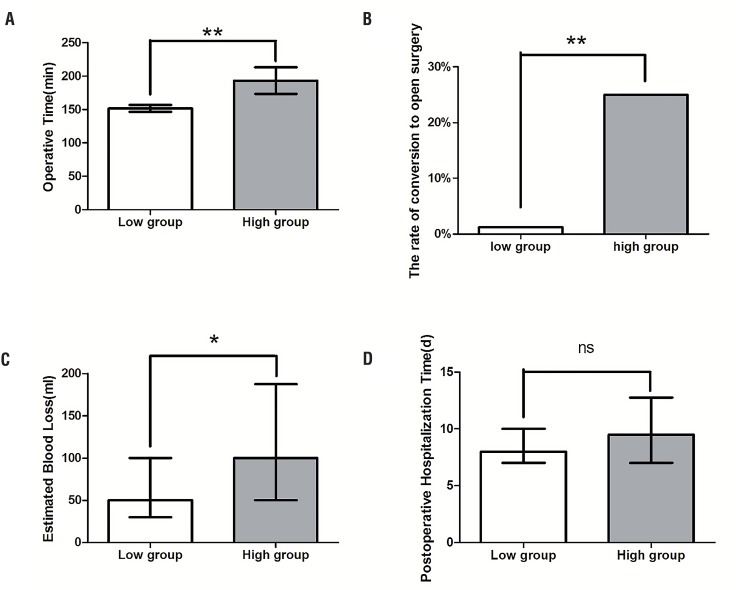
There were 83 and 12 patients in low (white bar) and high (gray bar) complexity groups respectively. And clinical outcomes analysis of patients treated with LRSN in two groups as the bar chart indicated. Operative time (A): OT of high complexity group was significantly longer than that of low complexity group (The mean OT was 193.2±69.3 min in high group and 151.9±46.3 min in low group, p = 0.008 by the two-sample t-test); the rate of conversion to open surgery (B): the high complexity group has a significantly higher rate of intro-operative conversion to open surgery (The rate of intro-operative conversion to open surgery was 25% in high group and 1.2% in low group, p = 0.006 by Chi-squared test); estimated blood loss (C): EBL of high complexity group was also significantly more than that of low complexity group (The median EBL was 100.0mL in high group and 50.0mL in low group, p = 0.034 by the Wilcoxon signed-rank test); and postoperative hospitalization time (D): no remarkable differences were observed between two groups relating to PHT (The median PHT was 8.0 d in high group and 9.5 d in low group, p = 0.187 by the Wilcoxon signed-rank test).

A total of 95 patients with benign kidney disease who underwent LRSN were enrolled in this study. They were divided into two groups on the basis of the HMUNS. The patient's baseline characteristics and perioperative outcomes are shown in Table-2. All kidney diseases were verified as benign by postoperative pathological examinations. The proportion of gender, mean age, surgical side, and mean BMI were comparable between the two groups (p >0.05). The OT (193.2±69.3 min vs. 151.9±46.3 min, p <0.05), EBL (100.0mL vs. 50.0mL, p <0.05), and rate of conversion to open surgery (1.2% vs. 25%, p <0.05) of the high-complexity group were significantly higher than in the low-complexity group, indicating higher HMUNS correlated with more difficulty and risk in LRSN (Table-2 and Figure-1). However, no remarkable differences were observed between the two groups relating to the PHT (8.0d vs. 9.5d, p >0.05) (Table-2). Postoperative fever, pain, bleeding, and incision infection, classified as CDC grade, were common complications, and there were no significant differences between the two groups (p >0.05) (Table-2).

**Table 2:** Patients baseline characteristics and peri-operative variables.

Variables	Low complexity		High complexity		*p* value
NO.patients	83		12		
Sum score	2-6		7-17		
Mean ages(year) x±SD	51.6±10.5		55.7±11.0		0.221
Sex(male:female)	33:50		6:6		0.542
Side(left:right)	45:38		4:8		0.176
Mean BMI(Kg/m^2^) x±SD	24.2±10.7		24.6±3.1		0.673
Mean OT(min) x±SD	151.9±46.3		193.2±69.3		0.008
Median EBL(mL)	50.0		100.0		0.034
Median PHT(d)	8.0		9.5		0.187
Rate of conversion	1/83(1.2%)		3/12(25%)		0.006
CDC	I-II	III	I-II	III	0.827
	81	2	12	0	

**BMI** = body mass index; **LRSN** = Laparoscopic retroperitoneal simple nephrectomy; **CDC**= Clavien-Dindo classification; **OT** = operative time; **EBL** = estimate blood loss; **PHT** = postoperative hospitalization time; SD = standard deviation

## DISCUSSION

As the technical aspects of LRSN can be affected by multiple variables, systematic scoring is needed based not only on patient-specific factors but also on the surgeon's experience. In doing so, to the best of our knowledge, this study represented the first comprehensive classification system to predict the potential complexity of LRSN. According to the HMUNS, the LRSN cases were divided into two groups, a low-complexity group (2-6 points) and a high-complexity group (7-17 points), respectively. A higher conversion rate, more EBL, and longer OT were detected in the high-complexity group than in the low-complexity group, suggesting that higher complexity and the likelihood of complications could be encountered in cases with higher HMUNS.

Perinephric and perihilar inflammation are the main concerns when performing LRSN (9, 10). Of the benign diseases that lead to the loss of kidney function, some, such as congenital anomalies, vascular pathologies, and systemic hypertension, were preferred by surgeons due to no or little inflammation. On the contrary, some diseases that cause severe inflammation and adhesion with adjacent structures are more challenging or are even considered “relative contraindications” (2). Much evidence has proven that LRSN can be performed successfully in some inflammatory diseases with concept innovations and technology improvements. In the present study, primary diseases such as renal tuberculosis (n=12), pyonephrosis (n=2), and ADPKD (n=1) were scored on a 2-, 2-, and 3-point scale. As the removal of tuberculous kidneys is technically demanding and always a challenge for urologists, currently, the data from only two recent studies including more than 50 patients addressing LRSN for tuberculous non-functioning kidneys yielded similar perioperative results regarding success rates (89.9% to 98.0%), operative times (107 min to 225 min), blood loss (50mL vs. 650mL), hospital stays (7 d to 14 days) and complications (only 1 case needed re-operation in 2 reports) (10, 11). These data indicate that LRSN is feasible for the treatment of non-functioning tuberculous kidneys. In addition to tuberculosis, the main causes of pyonephrosis are stone diseases, ureteropelvic junction obstruction, and diabetes mellitus. Hemal and Mishra reported that LRSN could be accomplished successfully in the majority of patients with pyonephrosis (88.5%, 46/51). The mean operative time and blood loss were 110 min (range 90-180) and 95mL (range 80-300), respectively. The mean hospital stay was 3.6 days (range 2-8) (11). Similar results were also observed by another study (12). These data suggest that LRSN is a reliable option for pyonephrotic non-functioning kidneys.

**Figure 2:**
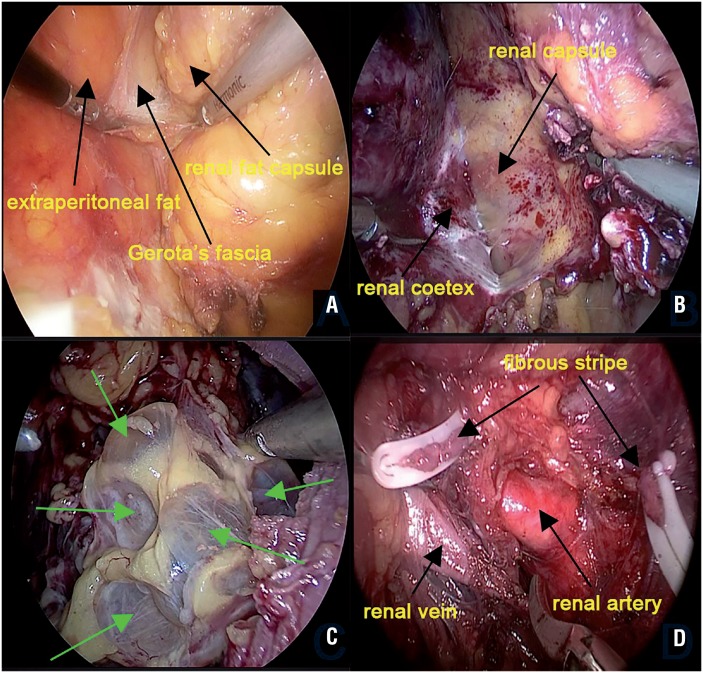
A) Mobilization kidney between Gerota's fascia and renal fat capsule; B) Mobilization kidney between renal fat capsule and the renal capsule or removing kidney with a subcapsular excision to avoid the adhesive parts; C) Arrows: cysts. Continuous volume reduction by cyst aspiration whenever possible in ADPKD; (D) Cutting off the sticky fibrous stripes which may contain minute vessels and adhesive lymph nodes linked closely with renal artery or vein around renal pedicle.

Because of substantial kidney volume, recurrent perirenal infection, bleeding, and percutaneous nephrostomy or surgical history, nephrectomy for ADPKD is an extremely challenging procedure. Laparoscopic techniques have been successfully used for bilateral synchronous or unilateral nephrectomy in ADPKD patients since 1996, and most surgeries are performed via transperitoneal routes (13–16). We found only two studies employing retroperitoneoscopic techniques in the PubMed database (17, 18). One study with 39 cases reported a mean operative time of 210.5 min, and another with 2 cases reported a mean operative time of 155 min. The mean intraoperative blood loss was 125mL, and no severe intraoperative complications occurred, but a postoperative retroperitoneal haematoma in 1 patient was managed conservatively with transfusion. The mean hospital stay was 3.6 days (18). In our study, only one LRSN was performed for ADPKD. Although no severe intraoperative or postoperative complications occurred, a longer operative time (375 min), more blood loss (300mL), and a longer postoperative hospital stay (6 days) resulted. Continuous volume reduction via cyst aspiration whenever possible (Figure-2), even splitting the kidney into parts if necessary, was vital. Considering the significant risk, LRSN should be performed for ADPKD only by experienced surgeons with adequate preoperative preparation.

Considering the impact of BMI and the learning curve on the LRSN procedure and outcomes, two components of our study were assigned 0-3 points and 1-2 points. As a multisystem chronic pro-inflammatory disorder, obesity may not only increase surgical difficulty, but also affect postoperative recovery (19). At present, BMI, one of the most common proxies for obesity, has been widely used for preoperative evaluation. The longer operative time for retroperitoneal nephrectomy was the major difference between normal BMI and obese patients (BMI >25) (20, 21). Another study found that BMI ≥30 significantly increased the conversion risk in LRSN for non-functioning renal TB (22). The significance of the learning curve is apparent for surgeons; therefore, diligent training and thorough knowledge are essential to gain sufficient surgical experience. Interestingly, laparoscopic retroperitoneal nephrectomy in extremely obese patients (BMI >40) (23) and nephroureterectomy in obese patients (BMI >30) (24) have been reported by experienced surgeons.

**Figure 3:**
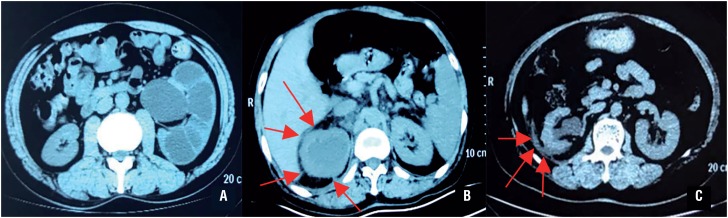
MAP score. A) type 1: no stranding; B) type 2: thin and mild stranding; C) type 3: diffuse, thick-banded stranding.

As kidney size and adherent perinephric fat (APF) can complicate the LRSN procedure by limiting the intact mobilisation of the kidney, the kidney volume (KV) and Mayo Adhesive Probability (MAP) scores (Figure-3) (5) were utilised to assess them and were included in our scoring system with different points. Renal width (W) was determined as the maximum width perpendicular to the renal length (L) on an identical slice of CT. Renal depth (D) was calculated as the maximum distance between the ventral side and the dorsal side of the kidney perpendicular to the renal length in a sagittal slice of CT. Kidney volume (V) was calculated using the formula KV=π/6×L×W×D. Published data showed that APF was present in 30% to 55.2% of patients who underwent partial nephrectomy and was associated with longer OT as a surrogate for surgical difficulty (5, 25). The MAP score is presently the only highly effective scoring tool for predicting intraoperative APF, and consists of the two most predictive factors, posterior perinephric fat thickness and stranding. When mobilising the kidney, especially in inflammatory conditions, identification of the safe layer is critical. The subcapsular layer (3) and underlying layer outside Gerota's fascia (5) have been reported as appropriate dissection planes for laparoscopy nephrectomy. Locating these layers and removing the kidney along them is ideal, however, adhesive fibrosis or APF makes the procedure difficult in some cases. As a result, any dissection layer between the kidney and retroperitoneum is feasible in our opinion. Once the safe layer proves difficult to identify in the surgical field, the surgeon should restart in another area and stay as far away from the retroperitoneum as possible. When this occurs, surgeon's patience and skill are decisive to avoid severe concomitant injury.

Compared with URS and PCNL, ESWL is thought to be less damaging to the kidneys, but based on the literatures and our experiences, shock waves induce significant damage to the renal and adjacent tissues and the extent of damage depends on the energy and the number of shock wave exposure (26). Therefore, multiple ESWL can increase perirenal infection (27) and injury, aggravating adhesions, making retroperitoneal nephrectomy more complicated.

Although no significant differences were observed between the two groups in terms of complications, severe bleeding and concomitant injury should be considered before LRSN, especially in cases with higher HMUNS. Controlling the renal vessels is crucial to reduce bleeding, and some technical and equipment improvements have been achieved in recent years. En bloc ligation of the renal hilum with or without Endo GIA^®^ has been reported to facilitate the procedure in some challenging cases (28, 29), but we prefer to dissect the renal artery and vein individually whenever possible to avoid rare renal arteriovenous fistula formation (30). Other complications such as fever, pain, incision infection, and others were also observed, but the differences between the two groups were statistically insignificant.

In summary, the complexity of LRSN is affected by multiple factors. We found that the operative time, intraoperative blood loss and conversion rate to open surgery of patients with high complexity were significantly higher than those with low complexity. We don't have to take minimally invasive surgery if preoperative evaluation shows a high score (≥ 7), Compared to laparoscopy, open surgery can better control intraoperative emergencies, such as bleeding and adhesion. The higher the score, the more difficult and complex the operation. Open surgery can remove adhesions more quickly and control intraoperative bleeding more effectively, thus reducing the difficulty of surgery and ending the operation as soon as possible. Therefore, we can directly choose open surgery if the preoperative score is higher. According to the HMUS system, the complexity of LRSN can be scored before surgery to predict the difficulty of surgery, so as to guide clinicians to reasonably choose surgical methods and treatment strategies (laparoscopy or open access).

## CONCLUSIONS

The HMUNS can effectively reflect complexity of LRSN before surgery. For some limitations consisting of a retrospective nature and sample size in our study could not be overcome, the HMUNS was just a relatively ideal scoring system, which should be more powerful, simple, and practical. Hence, well-designed trials with larger sample sizes are still needed to confirm our results.

## ABBREVIATIONS

APF: adherent perinephric fat
ADPKD: Autosomal Dominant Polycystic Kidney Disease
BMI: body mass index
CT: computed tomography
CDC: Clavien-Dindo classification
ESWL: extracorporeal shock wave lithotripsy
EBL: estimate blood loss
HMUNS: Harbin Medical University nephrectomy score
KV: kidney volume
LRSN: Laparoscopic retroperitoneal simple nephrectomy
MAP: Mayo Adhesive Probability
OT: operative time
PCNL: percutaneous nephrolithotomy
PHT: postoperative hospitalization time
SD: standard deviation
URS: ureterorenoscopy
